# Investigating the feasibility of selective anti-VEGF delivery to the retina using a microinvasive approach

**DOI:** 10.1007/s00417-025-06960-0

**Published:** 2025-09-18

**Authors:** Jared Ching, Mizuho Arai, Mayu Ooba, Misa Miyazato, Yumei Kobayashi, Shin Tanaka, Shohei Kitahata, Tatsuya Inoue, Maiko Maruyama-Inoue, Kazuaki Kadonosono

**Affiliations:** 1 Department of Ophthalmology and Microtechnology, Yokohama City University Medical Centre, Yokohama, Japan; 2 Department of Engineering Science, University of Oxford, Oxford, UK

**Keywords:** Microinvasive, Microneedle, Retinal surgery, Anti-VEGF, Endovascular cannulation.

## Abstract

**Background:**

Retinal therapeutics has been revolutionized by anti-vascular endothelial growth factor (VEGF) therapies, whereby intravitreal injection is the accepted delivery method. To date, the possibility of direct surgical delivery to the subretinal space or an endovascular approach has yet to be explored. Herein, we investigate the feasibility of a microinvasive approach for anti-VEGF therapies using microneedles as small as 49G in internal diameter.

**Methods:**

In vitro microinjections of commercially available anti-VEGF products, including broliucizumab, faricimab, aflibercept, and ranibizumab, were used to measure the consistency of 1µL droplet formation and needle occlusion thresholds. A validated fresh ex vivo porcine model used to examine microinjections of anti-VEGF solutions with fluorescent microbeads in the subretinal space and intravascular delivery. Spectral domain optical coherence (SD-OCT) imaging and histological retinal wholemounts were used to analyse the drug distribution.

**Results:**

In vitro, we find that of the commercially available formulations of aflibercept, brolucizumab, faricimab and ranibizumab, a 5% aflibercept solution and 2.5% ranibizumab solution can be reliably injected as 1µL droplets repeatedly through a microneedle. Using a fresh porcine ex vivo closed vitrecomy model, we demonstrate that ranibizumab admixed with fluorescent microbeads, can be reliably injected in ca. 25µL volumes into the subretinal space and endovascularly, without any risk of needle occlusion. We demonstrate using SD-OCT, retinal whole mounts and immunofluorescent microscopy, that direct retinal anti-VEGF delivery is feasible.

**Conclusions:**

We conclude that further study is warranted to determine whether such an approach has any advantages over the conventional approach of intravitreal injections in terms of treatment safety, efficacy and durability.

**Key messages:**

***What is known***

- Injections of anti-vascular endothelial growth factor (VEGF) therapies in the intravitreal 23 space are widely used to treat retinal diseases, requiring repeated and frequent 24 injections due to the limited durability of these therapies.
- New approaches to address this include implantable reservoir systems to reduce the 26 need for repeated injections.

***What is new***

- The feasibility of a microinvasive approach to deliver anti-VEGF therapies is explored 30 using microneedles as small as 49G.
- Low concentrations of the commercially available anti-VEGF products ranibizumab and 32 aflibercept can be injected into the subretinal space and intravascularly in an ex vivo 33 porcine model.
- These results warrant further study to understand if such approaches affect efficacy and 35 durability of anti-VEGF therapies.

## Introduction

Frequently presenting retinal diseases of the macula have been found to have significant vascular changes associated with vascular endothelial growth factor (VEGF), notably neovascular age related macular degeneration (nAMD), diabetic retinopathy, and retinal vascular occlusions [1, 2]. Following the discovery of the anti-VEGF chemotherapeutic bevacizumab (Avastin, Genentech, South San Francisco, CA, USA) that was first used in colon cancer, the first effective use of intravitreal anti-VEGF in nAMD was subsequently undertaken, representing a revolution for retinal therapy at the time [3, 4]. Since then, a number of novel anti-VEGF agents have been developed specifically for retinal disease, including ranibizumab (Lucentis, Genentech, South San Francisco, CA, USA), and aflibercept (Eylea, Regeneron, Tarrytown, NY, USA), which have been shown to improve visual acuity in up to 95% of treated patients [5–7]. The next generation of anti-VEGF agents have been shown to be more “durable” and are therefore able to be given at longer dosing intervals of 12 weekly and 16 weekly for brolucizumab (Beovu, Novartis, Basel, Switzerland) and faricimab (Vabysmo, Genentech, San Francisco, CA), respectively [8–11]. 

The currently available anti-VEGF products for intravitreal injection come as a pre-filled syringe or vial that needs to be drawn up into a syringe before delivery through a needle, most commonly 30 gauge or smaller, that is passed through the sterilized sclera 3.5–4.0 mm from the limbus depending on phakic status [12, 13]. Needles as small as 34 gauge have been shown to reduce pain scores in a randomized clinical trial [14]. Further, a meta-analysis confirmed that 30G needles or smaller can reduce pain, and their recommendation is to use as small a needle as possible [15]. Otherwise, direct retinal delivery of anti-VEGF has yet to be explored, partly because the intravitreal route is less invasive and does not require the more costly set up of vitrectomy surgery. However, the frequent need for repeat injections not only cumulatively increases the risk of sight threatening conditions, but significantly increases the number of outpatient attendances and ability of publicly funded hospitals to treat patients beyond Medical Retina [16]. The Port Delivery System (PDS) has been developed in order to avoid frequent intravitreal injections by enabling controlled release anti-VEGF into the vitreous cavity with infrequent procedures to refill the device with anti-VEGF required [17]. Unfortunately, the FDA approved PDS Susvimo was recalled in 2023 due to the frequency of partial dislodgement of the device (septum) following anti-VEGF refilling [18]. Most recently, the PDS has been re-introduced in Phase III clinical trials in the VELODROME study. As such, there is an urgent need to investigate alternative methods of anti-VEGF delivery. Currently, a direct trans-vitreal or intravascular approach to the retina has yet to be investigated for anti-VEGF delivery.

Given our groups’ experience with developing the first microfabricated microneedle of an estimated 47 and 48 gauge that can be used reliably to cannulate the retinal vasculature to treat vaso-occlusive diseases, we sought to explore minimally invasive approaches of direct retinal anti-VEGF delivery [19–21]. We hypothesized that selective anti-VEGF therapy may have a range of implications for treatment efficacy, frequency and offer a new avenue to explore novel forms of drug delivery. Herein, we present pilot data demonstrating the feasibility of minimally invasive anti-VEGF delivery to the retina.

## Methods

### Microscopy of microneedles

A Leica S9D stereomicroscope (Leica Microsystems GmbH, Wetzler, Germany) was used to measure the needle tips at a fixed zoom of 10X. All images were undertaken using a Flexacam C3 microscope camera (Leica Microsystems GmbH, Wetzler, Germany). All images were exported into Image J and analysed by zooming maximally to take measurements of the outer diameter, wall thickness and inner diameter.

### Electron microscopy of microneedles

All microneedles were stabilised on a stage using carbon tape and scanning electron microscopy was undertaken using a Keyence VE-8800. All images were undertaken using accelerating voltages at 0.8 kV or 2.0 kV.

### Anti-VEGF antibodies

**Fig. 1:**
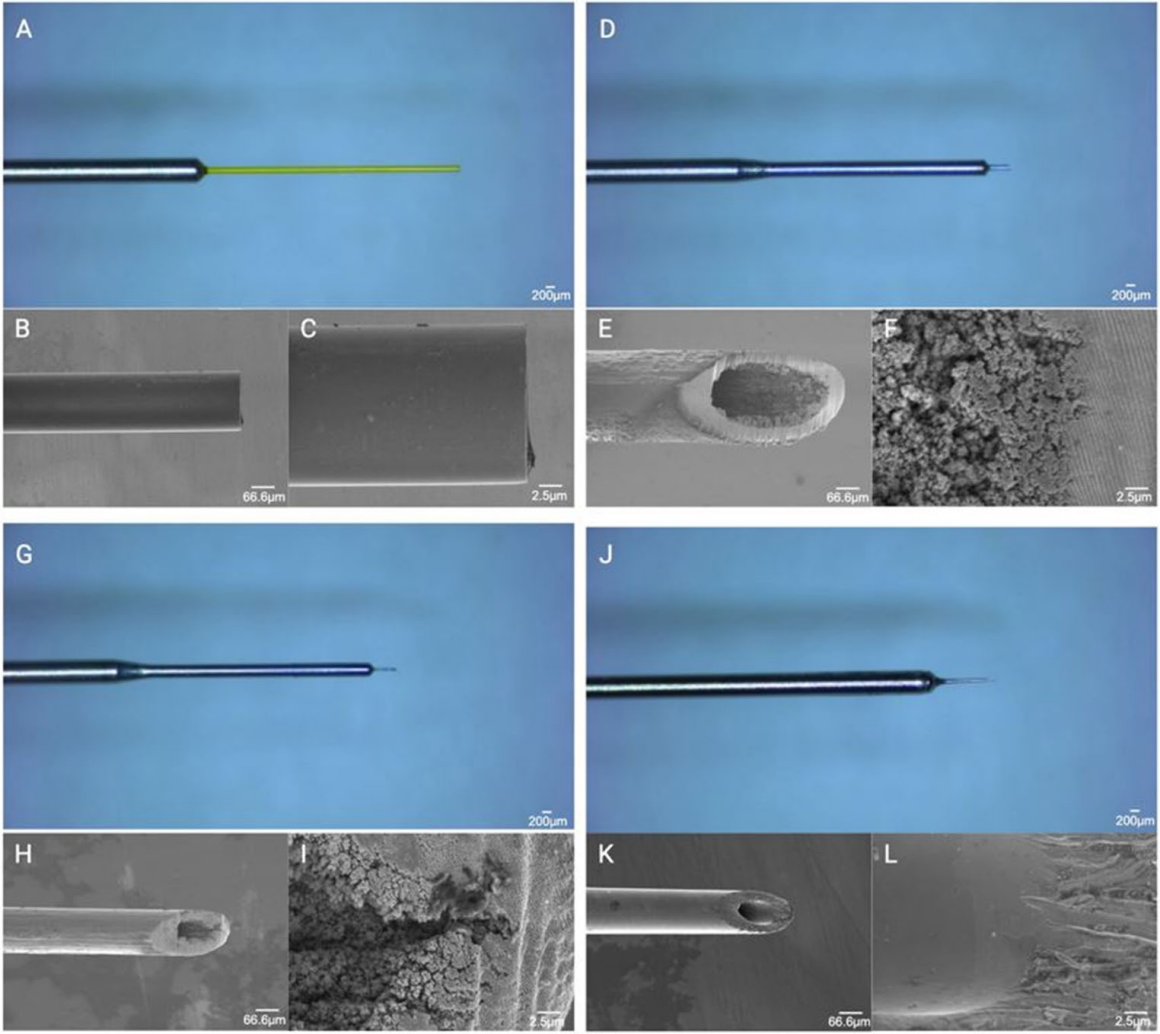
Minimally invasive retinal needles. Photomicrographs and corresponding electronic microscopy images of 25G/38G polytip (**A-C**), 38G/41G needle (**D-F**), 45G/49G needle (**G-I**), 43G/52G (**J-L**)

All antibody solutions were collected from the outpatient Medical Retina Clinic at the Yokohama City Unversity Medical Center, Yokohama, Japan (Table 1). Each antibody solution was diluted according to Table 2 and subsequently Table 3 using the ratio between the standardized intravitreal injection of 0.05mL to the average vitreous cavity volume of approximately 4mL [22]. Double distilled water (ddH_2_O) was used as the diluent as is it mimics water for injection used as an excipient in many anti-VEGF formulations and therefore theoretically reduces the risk of causing any significant issues such as protein denaturization or aggregation. We appreciate this may require validation studies prior to being licensed as a therapeutic but for the purposes of a feasibility study, this appeared to be a rational approach. A clean 50µL Hamilton syringe was washed with 100% ethanol then ddH_2_O and allowed to air dry. The following retinal needles were used: (1) MedOne Polytip^®^ cannula 25/38G x 5 mm tip (internal diameter 41G), (2) Tochigi Seiko microneedle 0.11 mm internal diameter, (3) MedOne Nano cannula 25G/48G, (4) Tochigi Seiko microneedle 0.05 mm internal diameter (Fig. 1; Table 4). We have taken measurements using microscopy and electron microscopy in a related study on microinvasive biopsy submitted elsewhere. The same convention, to reduce confusion, will be used according to these measurement as follows: 38G/43G needle, 38G/41G polytip, 45G/49G needle, and 43G/52G needle (Table 4). The anti-VEGF antibody solution of interest was then drawn up into the Hamilton syringe using a 25G needle before attaching the aforementioned microneedles. Each of the these needles were then attached to the primed 50µL Hamilton syringe and 5 x discrete 1µL droplets were dispensed onto a 96 well plate using a binocular dissecting microscope to ensure no physical trauma to the needle tip. Each droplet formed was measured with calipers to determine the largest basal diameter and photos taken with a Leica dissecting microscope. If the droplet formed was small or deficient, it was still detached onto the substrate surface and the process continued consecutively. This was then repeated twice before using a higher concentration of anti-VEGF. Between repeats and changes in anti-VEGF concentration, the needles were washed thoroughly with ddH_2_O using a 10mL Luer lock syringe, passing air to dry the inner lumen of the needle. All experiments were undertaken at room temperature and pressure. Needle block was defined as a complete inability to restore flow following withdrawal and injection attempts of ddH_2_O, air, 100% ethanol and following soaking in a warm water bath for 5 min. Blockage was not considered if needle trauma was encountered.

**Table 1:** Licensed anti-VEGF treatments commonly used at the department of ophthalmology and microtechnology, Yokohama City university medical center

Active drug	Brand name	Manufacturer	Molecular weight	Strength	Standard dose	Excipients
Brolucizumab	Beovu	Novartis	26kD	120 mg/mL (19.8 mg in 0.165mL pre-filled syringe)	6 mg in 0.05mL	Sucrose, Sodium citrate, Polysorbate 80, water for injection
Aflibercept	Eylea	Bayer	115kD	11.12 mg in 0.278mL	2.0 mg or 0.4 mg 8.0 mg	Sodium dihydrogen phosphate, sodium monohydrate phosphate, sodium choride, sucrose, polysorbate 80
Faricimbab	Vabysmo	Roche	149kD	120 mg/mL (28.8 mg/0.24mL)	6 mg in 0.05mL	L-histidine, Acetic acid 30%, L-methionine, polysorbate 20, sodium chloride, D-sucrose, water for injection
Ranibizumab	NA - Rabubizumb	Senju	48kD	10 mg/mL (1.65 mg/0.165mL)	0.5 mg in 0.05mL	(Lucentis - alpha-trhelose dihydrate, histidine hydrochloride, polysorbate 20, water for injection)

**Table 2:** Initial anti-VEGF dilutions to make up to 500µL

Dilution ratio	Final concentration (%)	Anti-VEGF volume	Double distilled water
84 in 84	100	500	0
53 in 84	75	315	185
42 in 84	50	250	250
21 in 84	25	125	375
4.2 in 84	5	25	475

**Table 3:** Revised anti-VEGF dilutions to make up to 500µL

Dilution ratio	Final concentration (%)	Anti-VEGF volume	Double distilled water
8.4 in 84	10	50	450
6.3 in 84	7.5	37.5	462.5
4.2 in 84	5	25	475
2.1 in 84	2.5	12.5	487.5
0 in 84	0	0	500

**Table 4:** Microneedle dimensions taken from microscopy and electron microscopy undertaken in a related study submitted elsewhere. Based on these measurements we estimate the internal and external gauge and therefore adopt a naming convention that includes the outer and inner estimate gauges: 38G/43G needle, 38G/41G polytip, 45G/49G needle, and 43G/52G needle

Needle (product names)	Internal diameter	Outer diameter	Wall thickness	Estimated internal gauge	Estimated outer gauge
38G Tochigi Seiko	0.06	0.11	0.04	43G	38G
41G Polytip	0.08	0.11	0.01	41G	38G
47G Tochigi Seiko	0.03	0.05	0.01	49G	45G
MedOne Nanoneedle	0.02	0.06	0.01	52G	43G

### Anti-VEGF and microbeads Preparation

The required anti-VEGF and concentration was first prepared before adding a 1:100 ratio of fluorescent microspheres. The fluorescent polystyrene microspheres were 0.2 μm in size with range of 0.190 to 0.210 μm (Suncoast Yellow, product reference: FSSY002, Bangs Laboratory Inc, Indiana, USA).

### Ex vivo selective surgical delivery of anti-VEGF

**Fig. 2:**
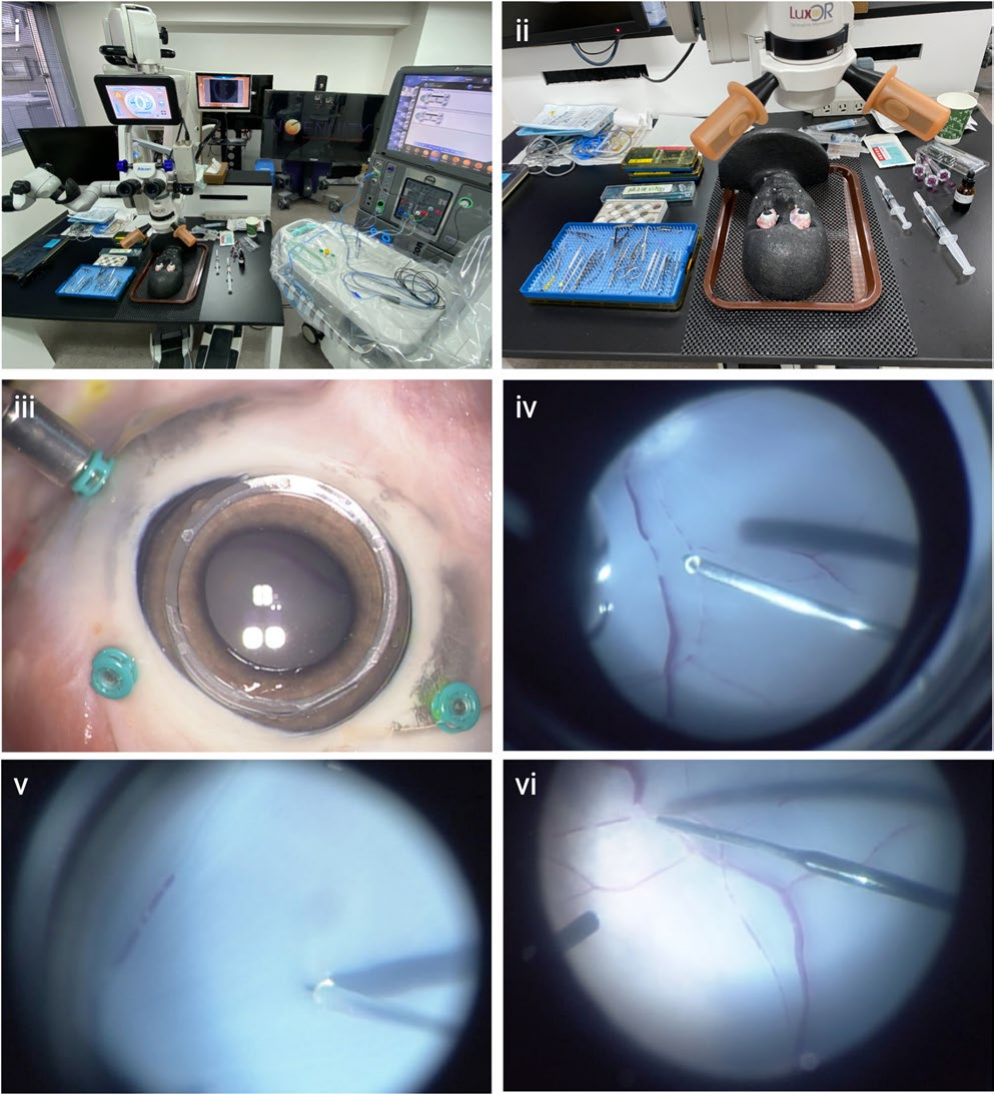
Ex vivo porcine vitrectomy model. (i-ii) Surgical microscope and Alcon Constellation Vision System set up with phantom head and freshly terminated pigs eyes mounted. (iii) 25G three port vitrectomy set up with contact lens over the cornea. Intraoperative view of vitrectomy (iv), subretinal injection (v) and intravascular injection (vi)

In order to confirm the feasibility of selective anti-VEGF delivery to the retina, we utilized an ex vivo pig eye model. Pig eyes were harvested from freshly terminated animals 3–6 months old from a local abattoir. The eyes were delivered on ice with the extraocular muscles and tissues intact and arrived at the laboratory within 2–4 h of termination. The pigs eyes were mounted onto a polystyrene phantom head and washed with double distilled water (ddH_2_O) (Fig. 2). Using the Alcon Constellation Vision System (Alcon, Texas, USA), a 25 gauge three port vitrectomy was performed on each pig eye. A vitrectomy contact lens was used as the posterior viewing system. A limited posterior vitreous detachment was performed in order to avoid retinal detachment and the posterior hyaloid membrane was peeled with end gripping forceps where necessary to clear posterior vitreous. The viscous fluid control (Alcon, Texas, USA) set was used to withdraw and inject the solution of anti-VEGF and microbeads. A MicroDose™ kit (MedOne, Florida, USA) was used to first aspirate the anti-VEGF and microbead solution through a 20 gauge cannula provided in the VFC pack, before exchanging this for the needle of interest and setting the Constellation to the inject mode. The extraction settings were set to a maximum of 650mmHg and the inject settings were set to a maximum of 30PSI and manually adjusted with the linear foot pedal as needed. As the porcine retina has no defined macula, an area of retina at the posterior pole away from the optic nerve in the region of the visual streak was selected in each eye for a subretinal injection of 50µL of anti-VEGF and fluorescent microbeads solution. Following this, four diathermy marks were made on surrounding attached retina, forming a square that permits triangulation of the injection site for focused histological analysis. Retinal endovascular injections of the anti-VEGF and fluorescent microbeads solutions was performed by puncturing a target vessel and gently injecting the solution of anti-VEGF and fluorescent microbeads. The end point was dilution and flush of the distal vessels. If a subretinal bleb formed instead, this was considered a failed endovascular cannulation and the eye discarded. To mark the injection site, four diathermy marks were made in the same fashion as for subretinal injections, however, diathermy of blood vessels was avoided to allow accurate analysis of the anti-VEGF and fluorescent microbead solution in distal regions of the retinal blood vessels. At the end of the procedure, the ports were left in situ before fundal imaging and no additional tamponade was utilised, such that the eye was partially filled with balanced salt solution and remaining vitreous.

### Imaging

Imaging was carried out using a Cirrus 5000 Optical Coherence Tomography (OCT) System (Zeiss, Oberkochen, Germany). The pigs eyes, while mounted in the phantom head, were imaged. The cornea was hydrated with balanced salt solution and viscoelastic. The corneal epithelium was removed if it was causing impairment of visualizing the retinal structures. The scanning sequences used included the macula cube and 5 raster scans.

### Histology

Following surgery, the enucleated pigs eyes were dismounted from the phantom head and an anterior chamber paracentesis was made at the limbus with a 22.5^o^ angled blade (Mani Inc., Tochigi, Japan) followed by two further stab incisions at the pars plana to facilitate ingress of fixative. The globes were then placed in 4% paraformaldehyde phosphate buffer solution (Nacalai Tesque, Kyoto City, Japan) at 4 °C overnight before immersion in 10%, 15% and 20% sucrose in PBS solution for 12 h each at 4 °C. Following this, the eyes were coated and immersed in optical cutting temperature medium and frozen using a metal plate over dry ice. The globes were then placed within an embedding cup before cryosectioning at 10 μm thickness using a Leica CM1950 Cryostat (Leica, Wetzler, Germany).

Whole mount retina preparations were then undertaken using previously described protocols [23]. En face imaging with fluorescent microscopy was then undertaken to visualize the anti-VEGF and microbead solution in the subretinal space, pre-retinal space (control) and intravascular space. The globes were then placed within an embedding cup, which was marked according to the location of the aforementioned diathermy marks on the retinal surface, before cryosectioning at 10 μm thickness using a Leica CM1950 Cryostat (Leica, Wetzler, Germany) 1 mm from the injection site. Sections were placed on a Super Frost coated glass slide (Matsunami Glass Ind. Ltd., Osaka, Japan), dried and stored at −20 °C before direct imaging or staining.

Hematoxylin and eosin staining was performed using a standard protocol, briefly, sectioned tissues were immersed in double distilled water (ddH_2_O) before applying haemotoxylin to cover the tissues for 3 min. The slides were rinsed twice with ddH_2_O before applying bluing reagent for 15 s. After rinsing with ddH_2_O, the slides were immersed in 100% ethanol and eosin was then applied. The slide was rinsed twice with 100% ethanol and then immersed in 100% ethanol before mounted with a cover slip.

### Statistical analysis

All statistical analysis was undertaken using GraphPad Prism 8.4.0 (455) for MacOS. Comparisons of anti-VEGF droplet size was undertaken using one way ANOVA, where statistical significance was defined as *p* < 0.05.

## Results

### Microneedle imaging

In order to characterize the dimensions and lumen topography, we undertook a combination of microscopy and scanning electron microscopy. The dimensions of the needle are described elsewhere in a related study examining microinvasive intraocular biopsy with imaging data with the cross-sectional measurements from this work summarized in Table 4. The lumen topography was not studied in the 38G/41G polytip as it was assumed the inner surface would match the outer surface, which was found to be smooth and free of irregularities (Fig. 1A-C). In comparison, the 38G/43G needle demonstrated an irregular topography that line the lumen at the bevel of the needle (Fig. 1D-F). The 45G/49G needle had a similar appearance to this and confirmed the relatively inconsistent nature of this (Fig. 1G-I). In contrast, the 43G/52G needle had a smoother appearance with the absence of any significant irregularities at the bevel, possibly reflecting the difference in manufacturing methods (Fig. 1J-L) to the other two microneedles.

### Microlitre delivery of anti-VEGF in vitro

In order to investigate whether a very small volume of anti-VEGF can be delivered selectively to a specific region in the retina we utilized manually operated syringes to assess the feasibility of this. We chose 4 microinvasive needles to test this hypothesis using glass micro syringes commonly used in animal studies (see Materials and Methods). We found that volumes larger than 1µL caused backpressure in the smallest microneedles, creating inconsistent droplets. As such, we assessed the ability of each needle to dispense five consecutive 1µL droplets and this was repeated twice with a high pressure washout of the needle with double distilled water between experiments. Droplets were detached via contact on a 96 well plate lid and imaged. The largest diameter of the droplets were recorded. We further rationalized that different anti-VEGF treatments have different concentrations and molecular weights. We therefore tested each of the anti-VEGF products as listed in Table 1.

Our initial investigation utilized brolucizumab concentrations of 25%, 50%, 75% and 100% as calculated in Table 3. Given that we had access to higher quantities of excess of brolucizumab from clinic and this has the lowest molecular weight of the anti-VEGF therapies available in our setting, we started our initial experiments with this drug. We found that when using the 38G/41G Polytip, this worked well up to 100% concentration but when using the 38G/43G microneedle, total blockage occurred mid-way through 75% concentration of brolucizumab (data not shown). Further, we found that both the 45G/49G microneedle and MedOne Nanoneedle blocked at 25% concentration of brolucizumab. On this basis, we amended the anti-VEGF concentrations to a lower range of concentrations from 2.5 to 10% (Table 2).

**Fig. 3:**
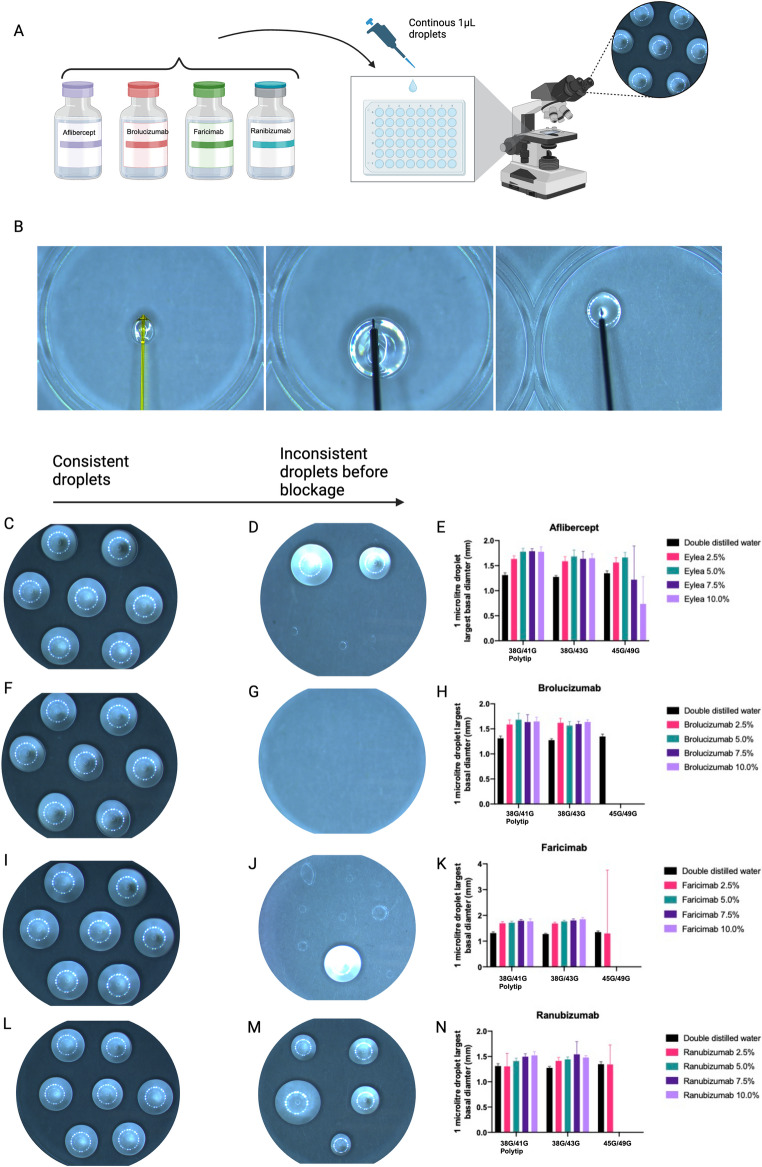
(**A**) Schematic demonstrating workflow: each anti-VEGF is diluted accordingly and then 1µL droplets are formed continuously under microscopy until blockage occurs. (**B**) Image from 1µL droplets being injected onto the surface of a 48 well plate, from left to right, the 38G/41G polytip, 38G/43G needle and 45G/49G needle. Photomicrographs with brightfield photographs demonstrating 5 to 6 consecutive droplets that were consistent without blockage (**C, F, I, L**). Finally, droplet size variability occurred leading up to the needle blockage (**D, J, M**), where brolucizumab 2.5% totally occluded the 45G/49G microneedle and hence no droplets could be expelled. Graphs comparing droplet size between different concentrations of anti-VEGF and needles (**E, H, K, N**)

With these revised lower drug concentrations between 0 and 10% we found that for both the 38G/41G Polytip and 38G/43G microneedle, the 1µL droplets were consistently formed without any significant deviation between repeats in all drugs tested (Fig. 3; Table 5). There was no statistically significant difference between the droplet sizes for aflibercept and brolucizumab, however a statistically significant difference was found for faricimab (*p* = 0.0187) and ranibizumab (*p* = 0.0027).

**Table 5:** Mean largest basal diameter (mm) of 1µL droplets continuously expelled out of different microneedles with double distilled water (control) or solutions of anti-VEGF from 2.5–10.0%. This table represents the average and standard deviation (SD) of three separate experiments with at least 5 droplets expelled and measured for each

Anti-VEGF		Double distilled water (Control)		2.50% anti-VEGF solution		5.00% anti-VEGF solution		7.50% anti-VEGF solution		10.00% anti-VEGF solution	
Aflibercept	Needle	Mean largest diameter (mm)	SD	Mean largest diameter (mm)	SD	Mean largest diameter (mm)	SD	Mean largest diameter (mm)	SD	Mean largest diameter (mm)	SD
	38/41G polytip	1.31	0.05	1.63	0.06	1.78	0.06	1.79	0.05	1.79	0.05
	38G/43G needle	1.27	0.03	1.59	0.09	1.68	0.13	1.64	0.15	1.65	0.09
	45/49G needle	1.35	0.05	1.56	0.10	1.66	0.10	1.22	0.67	0.74	0.54
Brolucizumab	38/41G polytip	1.31	0.05	1.59	0.09	1.67	0.74	1.43	0.80	1.19	0.73
	38G/43G needle	1.27	0.03	1.62	0.09	1.57	0.08	1.60	0.05	1.64	0.04
	45/49G needle	1.35	0.05	Blocked	N/A	N/A	N/A	N/A	N/A	N/A	N/A
Faricimab	38/41G polytip	1.31	0.05	1.69	0.07	1.72	0.05	1.79	0.05	1.77	0.10
	38G/43G needle	1.27	0.03	1.68	0.05	1.76	0.05	1.80	0.06	1.64	0.83
	45/49G needle	1.35	0.05	1.30	2.46	Blocked	N/A	N/A	N/A	N/A	N/A
Ranibizumab	38/41G polytip	1.31	0.05	1.30	0.26	1.41	0.06	1.50	0.06	1.52	0.07
	38G/43G needle	1.27	0.03	1.41	0.07	1.44	0.05	1.54	0.25	1.48	0.04
	45/49G needle	1.35	0.05	1.34	0.38	Blocked	N/A	N/A	N/A	N/A	N/A

The 45G/49G needle blocked when attempting to inject concentrations of 5% faricimab and ranibizumab (Fig. 3E & K). We found that even concentrations as low as 2.5% brolucizumab blocked the 45G/49G needle (Fig. 3H). We demonstrate that aflibercept can be injected consistently at a concentration of 5% and less reliably with a higher standard deviation at 7.5% and 10% concentration (Fig. 3N). Prior to blockage of the 45G/49G needles, we found that 1µL droplets were formed inconsistently (Fig. 3D, G, J, M). In contrast, the 38G/41G polytip and 38G/43G needles did not block or lose consistency in injecting 1µL droplets.

### Ex vivo selective surgical delivery of anti-VEGF

**Fig. 4:**
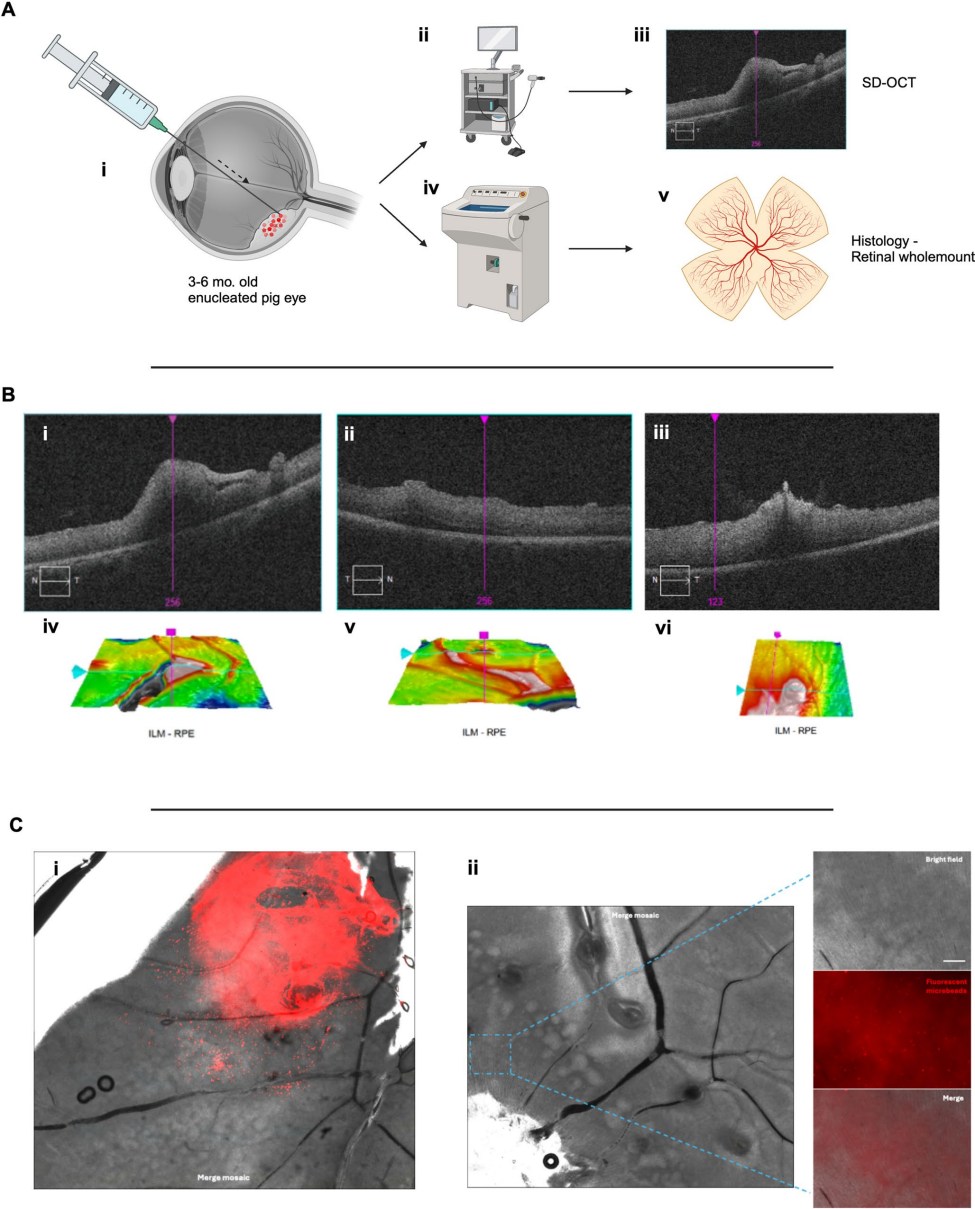
(**A**) Schematic demonstrating experimental process: (i) closed vitrectomy porcine model with subretinal injection of 5% aflibercept and 1:100 fluorescent microbead solution, (ii-iii) spectral domain optical coherence tomography (SD-OCT), and histological processing for retinal wholemount preparation. (Bi-iii) SD-OCT images showing the puncture size of microneedle delivered 5% aflibercept and 1:100 fluorescent microbead solution in the subretinal space focused on the puncture site demarcated by diatheramy marks. (Biii) demonstrates the presumed microneedle puncture site with a hyperreflective foci present on the inner retina. (Biv-vi) 3D reconstructions of corresponding SD-OCT images. (Ci) Posterior vitreous cortex injection of 5% aflibercept and 1:100 fluorescent microbead solution as a positive control in a retinal wholemount preparation. (Cii) Retinal wholemount following a subretinal injection of 5% aflibercept and 1:100 fluorescent microbead solution, showing the site of injection in the zoom panel. This zoom panel demonstrates that the fluorescent microbeads are confined to the subretinal space, where no clear pre-retinal microbeads are visualized through the different z-planes (data not shown). The zoom panel represent the maximum zoom project of the z-stacks taken in this region

Subretinal delivery of anti-VEGF was investigated by using a solution of 5% aflibercept mixed with fluorescent microbeads and injected into the subretinal space before SD-OCT imaging and histology (Fig. 4). Brolucizumab, faricimab and ranibizumab was avoided given the less reliable delivery profile found using the microneedle. First, to examine the immediate ex-vivo appearance of the microbeads in the vitreous, we injected the aflibercept-microbead solution first in the posterior vitreous cortex as a positive control (Fig. 4Ci) and then in the subretinal space (Fig. 4Cii). Surgically, when using the 38G/41G Polytip, 38G/43G needle, and 45G/49G microneedle, there was no impairment in delivering approximately 25µL of the aflibercept-microbead solution in the three repeats for each condition. No needle blockages occurred and no significant reflux was seen after withdrawing the needles from the subretinal space. Histological analysis demonstrated that the positive control demonstrates a densely fluorescent aflibercept-microbead solution at the pre-retinal space (Fig. 4Ci). Regions of subretinal injection were examined using a retinal whole mount, finding that the fluorescent solution was not easily visible at the injection site (Fig. 4Cii), where histological sections did not appear to have any fluorescent microbeads present (data not shown). After this was repeated a number of times, it was concluded that the microbeads are washed away during the preparation processes, which was confirmed in related experiments examining retinal pigment epithelial (RPE) cell transplantation.

**Fig. 5:**
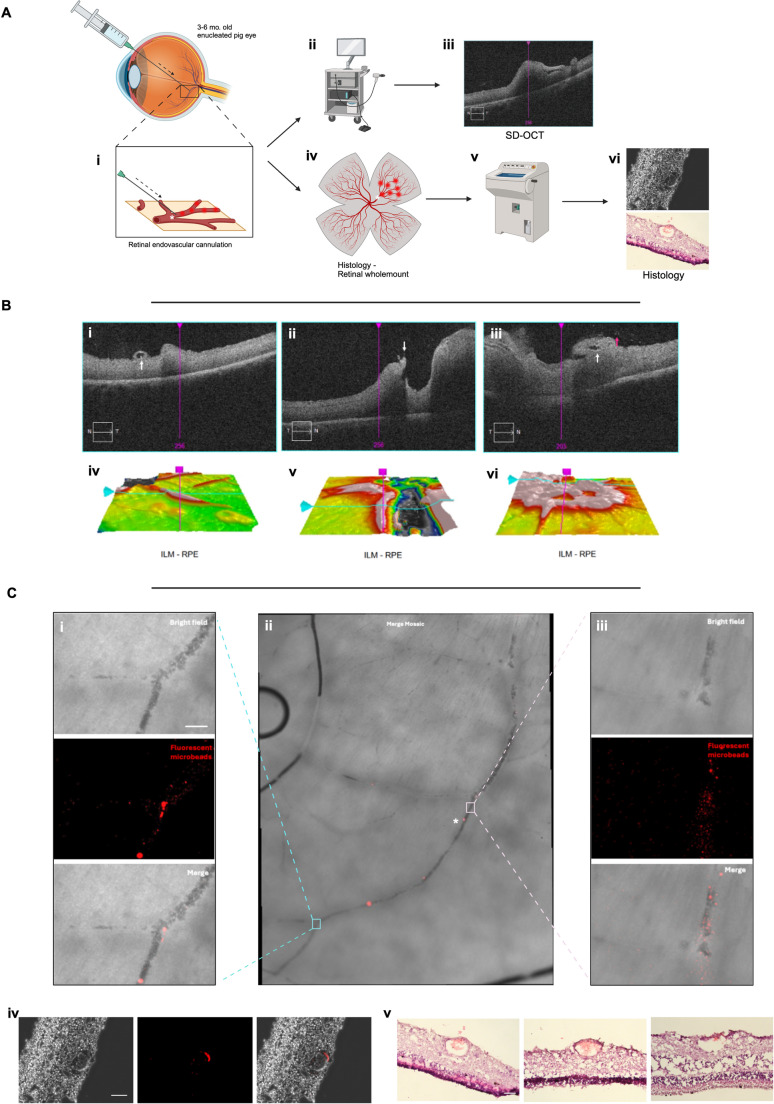
(**A**) Schematic demonstrating experimental process: (i) closed vitrectomy porcine model with endovascular injection of 5% aflibercept and 1:100 fluorescent microbead solution, (ii-iii) spectral domain optical coherence tomography (SD-OCT) scans are performed immediately after, followed by (iv) retinal whole mount preparation and imaging, and finally (v) targeted cryosectioning and (vi) staining. (**B**) SD-OCT images demonstrating the presence of (i) intermittent hyperreflective foci that may represent conglomerated fluorescent microbeads (white arrow), (ii) the needle injection site and focal fluorescent microbead reflux (white arrow), and (iii) perivascular (red arrow) and intravascular hyperreflective foci (white arrow). (Biv-vi) 3D reconstructions of corresponding SD-OCT images. (**C**) Histological images demonstrating a (ii) retinal whole mount mosaic image following the course of a major retinal vessel that was cannulated with a microneedle and injected with 5% aflibercept and 1:100 fluorescent microbead solution. (Ci) demonstrates a zoom panel demonstrating a distal vascular bifurcation with microbeads present with the lumen of the retinal vessel and along the inner wall of the vessel. (Ciii) The injection of the microneedle demonstrating vascular flow of the 5% aflibercept and 1:100 fluorescent microbead solution through the vessels. (Civ) Immunofluorescence imaging of a section at the corresponding site marked with (*) on Cii, showing that the fluorescent microbeads are located within the vessel. (Cv) Hematoxylin and eosin sections within 60 μm of Civ, confirming the anatomy of the retinal vessel and presence of blood within the vessel lumen. Immunofluorescent panels represent the maximum zoom project of the z-stacks taken in this region (Ci, iii, iv)

In order to examine the feasibility of delivering anti-VEGF via an endovascular route, the aflibercept-microbead solution was injected via the 45G/49G microneedle in the retinal vasculature (Fig. 5). SD-OCT showed the presence of intermittent hyperreflective foci that may represent conglomerated fluorescent microbeads (Fig. 5Bi), the needle injection site and focal fluorescent microbead reflux (Fig. 5Bii), and perivascular and intravascular hyperreflective foci (Fig. 5Biii).

## Discussion

We rationalized that in order to characterize the flow of anti-VEGF through a micro-needle, a series of dilutions would be necessary given that high concentrations of antibodies are known to have greater viscosities, affecting their flow properties [24]. Further, the current standard of intravitreal injections is 0.05mL, which is a very small volume relative to the estimated volume of the vitreous cavity of 4.0-4.4mL [22]. As such, when considering localized minimally invasive therapeutic delivery, such high monoclonal antibody concentrations may not be necessary or feasible through a very small gauge needle. Given the dilution effect 0.05mL of antibody solution dispersing in approximately 4mL of vitreous, with a high water content, we diluted the anti-VEGF antibody solution as low as 2.5% to gain insights into the feasibility of delivery such therapeutics in a minimally invasive and selective way. We chose 5% as a crude lower bound as the average macula is thought to account for approximately 4% of the total retinal area of 1,065m^2^ [25, 26]. We show that aflibercept can be delivered reliably at this concentration with all needles used in the present study, including the 45G/49G microneedle, but not the 43G/52G microneedle. Further, we confirm that the 38G/41G polytip and 38G/43G needle can dispense 1µL of all anti-VEGF agents used in this study up to 10.0% concentration.

During this study we were surprised to find that blockages occurred with the 43G/52G needle when using ddH_2_O. Our analysis of possible reasons for this included potential contaminants in our syringes or lubricating silicone oil in syringes, which has been found to cause uveitis in intravitreal injections [27]. We therefore attempted to address this by cleaning the Hamilton syringes used and using silicone oil free syringes, which we utilized the MicroDose SF (MedOne, Florida, USA). We found that this was not contributory as, blockages continued to occur with new 43G/52G microneedles using silicone oil free syringes. Phosphate buffer solution (PBS) and ethanol absolut were trailed, where it was found that neither caused any microneedle blockages. After clarifying any source of inadvertent needle blockage, the data we present was generated from using the same conditions throughout, i.e. the same type of Hamilton syringe was used with regular washing with ethanol, which gives us confidence that any blockages we encountered was due to the anti-VEGF solution used. We surmise that a possible issue with the 43G/52G needle is the manufacturing technique used as it appears the lumen size may vary between batches or even individual needles. We could not confirm this directly, however, given the variability of blockages that occurred throughout our experiments, we can only hypothesize from the observation that these needles may be manufactured in similar vain to pulled micropipettes for patch clamping neural cells [28]. Such methods are known to result in variations in lumen size, requiring measurement to confirm the size is within 15% of the target diameter, and whilst SEM is the gold standard to such measurements, more efficient means have been investigated [29]. 

The viscosity of different anti-VEGF formulations are affected by numerous factors, including high concentrations above 100 mg/mL [30], which applies to brolucizumab and faricimab, both being supplied as 120 mg/mL solutions commercially. This can lead to highly viscous antibody solutions with high levels of protein-protein aggregations as a result of reduced intermolecular distances [31]. We did not pursue measuring viscosity of the different anti-VEGF formulations but instead investigated whether reducing the concentration of these therapies made a difference in microneedle delivery. We found that concentrations of brolucizumab and faricimab above 2.5% were compromised and led to inconsistent drug delivery or needle blockage. In comparison, aflibercept (11.2 mg/mL) could be delivered reliably using a concentration of 5% and ranibizumab (10.0 mg/mL) 2.5%. This indicates that it is not be antibody concentration alone that affects microneedle delivery since aflibercept and ranibizumab are very similar in concentration.

Using SEM, we found that the inner lumen of the 43G/52G needle was smooth in comparison to the 45G/49G and 38G/43G needles. Again, this difference will be related to the manufacturing processes and materials and alloys used. The 45G/49G needle is fabricated using a proprietary three stage process that results in a very different shaft profile to the 43G/52G needle. While the 43G/52G needle tapers along its shaft, the 45G/49G needle has a straight shaft that is attached to a hollow tube with a small taper between the two components. Further, our cross-sectional measurements (data presented in a related paper elsewhere) demonstrate that the 43G/52G needle has a 10 μm smaller lumen than the 43G/52G. This can affect fluidics and we demonstrate this using the Reynolds number and the Hagen-Poiseuille equation to estimate differences in shear stress, data presented elsewhere in a related study on microinvasive cell therapy. We predict that the combination of fluidics dictated by the differences in lumen sizes, inner surface topography, and needle shaft shape are important considerations for microneedle blockages when delivering different concentrations of anti-VEGF drugs.

Subretinal gene therapy to treat Lebers Congenital Amaurosis has been shown to be safe and effective, using the 38G/41G polytip for surgical delivery, which is well established in the field [32, 33]. Further, subretinal tissue plasminogen activator has been used in numerous studies with variable results, where the TIGER Trial aims to clarify its therapeutic role in presentations of submacular haemorrhage, using the same delivery cannula [34]. Finally, induced pluripotent stem cell derived RPE cells have been delivered as cell suspensions with such cannulae but there is still room for improvements given that cell reflux through the retinal wound is thought to cause epiretinal membranes [35]. To our knowledge, subretinal anti-VEGF therapy has not been investigated in the context of selective retinal therapy or via the retinal intravascular route. In the field of cancer therapeutics, the anti-VEGF bevacizumab (Avastin^®^) has an established role in a certain cancers when given systemically. Avastin has also been administered intravenously for age related macular degeneration previously, demonstrating efficacy [36], however the intravitreal route has become the preferred route of administration due to the preferred safety profile [37]. Herein, we suggest that the selective subretinal and endovascular routes for anti-VEGF may have a role in ophthalmic practice in the future, where the durability and efficacy of these agents may be modulated without significantly changing the risk profile of administration compared to the intravenous route. However, this will depend on assessing this in animal models in the first instance, which the present study does not address.

Endotamponade tamponade with air, gas or silicone oil is known to aid retinal detachment, however, in the context of drug delivery and submacular haemorrhage therapeutics, it is known that subretinal substances can be displaced away or towards the targeted retina. In gene therapy, it has been shown that adenoviral associated virus (AAV2) delivered in the subretinal space, transduces green fluorescent protein away the subretinal bleb air endotamponade is utilised [38]. A similar effect is seen with gas endotamponade when treating submacular haemorrhages [39]. For the present feasibility study, we did not explore these effects but this will be an important consideration depending on the therapeutic target.

Furthermore, subretinal bleb morphology, including shape, height, and anatomical position have been shown to play an important role in subretinal therapies in terms of potential effectiveness and risk of complications [40]. Retinal stretch and reflux through the retinotomy has been calculated to potentially occur more frequently using a spherical cap formula, which is also relevant when considering subretinal anti-VEGF therapy to minimise iatrogenic injury through excessively high blebs with large volumes [41]. However, these characteristics were not considered in detail in the present feasibility study owing to differences in porcine and human retinal anatomy, for example the pig retina has a visual streak rather than a macula. Further, it was envisaged a microinvasive would permit numerous small and targeted subretinal blebs with potentially self-sealing retinotomies, which would be less like to be associated with complications relating to retinal stretch. The anatomical effect of subretinal injection location in the live porcine retina has been characterised in the context of gene therapy, providing important considerations for future studies on superselective subretinal injections of anti-VEGF [42]. We predict that with smaller subretinal blebs and retinotomies formed by microneedles, the risk of reflux can be mitigated, improving the safety profile of such approaches, including microinvasive delivery of gene therapy. Altogether, such considerations will be taken into account in our future studies that we aim to use disease models to assess safety and efficacy of a microinvasive therapeutic approach using anti-VEGF agents, gene therapy and other therapeutic agents.

Herein we suggest that retinal endovascular delivery of anti-VEGF is feasible based on prior nonrandomised clinical trials using systemic anti-VEGF (bevacizumab), which showed efficacy in AMD and non-AMD related choroidal neovascularisation but can be associated with hypertension [36, 43]. We surmised therefore, that local superselective treatment at the level of the retinal vessels may enhance efficacy through greater bioavailability and direct tissue perfusion, with a lower incidence of systemic complications. Reports to date demonstrate few surgical complications associated with these procedures including vitreous haemorrhage requiring repeat surgery in 1 out of 10 patients [21], with not systemic complications reported [20]. However, it is acknowledged that the paucity of studies and small sample sizes in available studies make it challenging to fully assess the risk profile, additionally given the pro-thrombotic nature of some anti-VEGF agents, this may cause thrombus formation theoretically. As such, the ideal anti-VEGF, concentration and formulation would need to be established in live animal studies before translating this. It will also be necessary to determine complications such as embolic disease, retinal detachment, raised intraocular pressure, improper vascular cannulation or perforation, and changes in intra-ocular pressure. Optimising the safety profile of such procedures is ongoing with multiple surgical robots in development [44–47]. As such, there are likely to be technical and pharmaceutical challenges that need to be addressed in order for superselective anti-VEGF therapy to be realised.

Limitations of this study are the use of ex vivo fresh pigs eyes and the lack of live animal studies to determine safety and efficacy. Previously, it has been shown that subretinal anti-VEGF may be effective in treating submacular haemorrhage, however, there is a paucity of clinical trial data to validate this approach [48]. Since the present study describes the use of lower concentrations of anti-VEGF, this may alter efficacy and durability. However, as it is uncertain how anti-VEGF products licensed for intravitreal delivery affect the retina when delivered in the subretinal space, we believe this deserves more detailed study with disease models. Further, we have found in our related work on microinvasive biopsy that retinotomies created by standard approaches to deliver subretinal therapy, have a propensity to leak. As such, it is uncertain how much anti-VEGF is retained in the subretinal space and also since gas tamponade is used to assist subretinal haemorrhage displacement this may affect the subretinal bleb, concentration of subretinal anti-VEGF and dispersion of the subretinal drug. In contrast, using a microneedle, we find that reflux of fluorescent microbeads is significantly reduce or completely avoided in a subretinal tumour model (data published elsewhere). Therefore, in order to translate this, the pharmacokinetics, formulation, and mechanisms by which the subretinal bleb of anti-VEGF is affected, need to be studied carefully.

Finally, novel ways to enhance long term delivery to avoid frequent intravitreal injections of anti-VEGF have been developed, including the PDS [18] and more recently, in situ forming hydrogels [49]. Such techniques, although more invasive, may have a role in reducing the need for repeat treatments and offer alternative strategies in challenging cases. We envisage that minimally invasive vitrectomy approaches that simply aim to inject therapeutic substances without performing a vitrectomy, may become an option in the future, in a similar vain to undertaking vitrectomy-assisted choroidal biopsies or potentially akin to vitrectomy-sparing subretinal gene therapy [50, 51]. 

## Conclusions

We demonstrate that it is feasible to deliver low concentrations of anti-VEGF formulations through microneedles using in vitro and ex vivo studies. We demonstrate that high concentration anti-VEGF formulations such as brolucizumab and faricimab may not have a favourable viscosity or other properties for selective retinal delivery, causing microneedle blockages. Our ex vivo experiments confirm that it is feasible to deliver 5% aflibercept to the subretinal space and endovascularly by using fluorescent microbeads to confirm this.
